# Letter to the Editor: Orthostatic Reactivity in Patients with Ischemic Stroke in the Chronic Period. OA Maced J Med Sci. http://dx.doi.org/10.3889/oamjms.2015.090

**DOI:** 10.3889/oamjms.2015.094

**Published:** 2015-08-21

**Authors:** Danche Vasileva

**Affiliations:** University “Goce Delchev”, Faculty of Medical Sciences, Shtip, Republic of Macedonia

**Keywords:** Orthostatic reactivity, Kinesitherapy, Neurorehabilitation, Neurodevelopment treatment, Ischemic stroke, Chronic period

**Respected Editor-in-Chief**,

I am writing you about our publication Orthostatic Reactivity in Patients with Ischemic Stroke in the Chronic Period, published OnlineFirst in OA Maced J Med Sci on August 01, 2015 [1].

Tables and Figures are not adequate for this publication, because they are from the previously published paper Orthostatic Reactivity in Patients with Diabetic Neuropathy. OA Maced J Med Sci. 2014 Jun 15; 2(2):244-248 [2].

I am sending you correct Tables and Figures and ask you to correct them, if possible. Previous Tables and Figures were attached by mistake.

I would be very grateful if you can correct them.

Danche Vasileva,

University “Goce Delchev”,

Faculty of Medical Sciences,

Shtip, Republic of Macedonia

**Competing Interests:** The authors have declared that no competing interests exist.

**Respected Editor-in-Chief,**

I am writing you about our publication Orthostatic Reactivity in Patients with Ischemic Stroke in the Chronic Period, published OnlineFirst in OA Maced J Med Sci on August 01, 2015 [1].

Tables and Figures are not adequate for this publication, because they are from the previously published paper Orthostatic Reactivity in Patients with Diabetic Neuropathy. OA Maced J Med Sci. 2014 Jun 15; 2(2):244-248 [2].

I am sending you correct Tables and Figures and ask you to correct them, if possible. Previous Tables and Figures were attached by mistake.

I would be very grateful if you can correct them.

1. Vasileva D, Lubenova D, Mihova M, Grigorova-Petrova K, Dimitrova A. Orthostatic Reactivity in Patients with Ischemic Stroke in the Chronic Period. OA Maced J Med Sci. http://dx.doi.org/10.3889/oamjms.2015.090
2. Orthostatic Reactivity in Patients with Diabetic Neuropathy. OA Maced J Med Sci. 2014 Jun 15; 2(2):244-248. http://dx.doi.org/10.3889/oamjms.2014.039

Danche Vasileva,
University "Goce Delchev",
Faculty of Medical Sciences,
Shtip, Republic of Macedonia

**Table 1: Mean values, standard deviation, the highest and lowest values of descriptive characteristics by: age, weight and height of the patients studied, assessments of the stage of functional recovery according Brunnstrom and the test for spasticity according Ashworth, at the beginning of the study**

| Parameters | Age | Weight | Height | Brunnstrom - upper limb | Brunnstrom - lower limb | Ashworth - upper limb | Ashworth – lower limb |
|---|---|---|---|---|---|---|---|
| $X \pm S_D$ | 63.2±8.8 | 77.9±10.1 | 169.2±6.4 | 4.2±0.7 | 4.8±0.6 | 1.6±0.6 | 1.1±0.5 |
| Xmax | 76 | 101 | 182 | 5 | 6 | 3 | 2 |
| Xmin | 40 | 60 | 152 | 3 | 4 | 1 | 0 |

**Table 2: Mean values, standard deviations, the largest and smallest data values of heart rate during orthostatic test in two (SOR and HOR) study groups before and after kinesitherapy distributed in classification by Thulesius**

| Parameter | Groups | SOR at beginning | 10th day | 1st month | 3rd month | HOR at beginning | 10th day | 1st month | 3rd month |
|---|---|---|---|---|---|---|---|---|---|
| Lying position 1 1 min | | 81.4±6.1 | 79.5±5 | 78.5±5 | 78.5±5 | 85.6±9.6 | 84.6±6.4 | 83.3±6.4 | 83.3±6.4 |
| | Xmax | 88 | 88 | 88 | 88 | 96 | 92 | 90 | 90 |
| | Xmin | 70 | 72 | 70 | 70 | 65 | 72 | 70 | 70 |
| 5 min | | 80±5.1 | 77.5±4.8 | 76±4.9 | 76±4.9 | 83±8.8 | 82±6.6 | 79.5±5.7 | 79.5±5.7 |
| | Xmax | 86 | 85 | 84 | 84 | 94 | 90 | 88 | 88 |
| | Xmin | 72 | 70 | 69 | 69 | 63 | 68 | 68 | 68 |
| 10 min | | 79.6±4.9 | 77.1±4.3 | 75.6±5.1 | 75.6±5.1 | 82.4±9 | 81.6±7.4 | 79.1±5.9 | 79.1±5.9 |
| | Xmax | 86 | 80 | 84 | 84 | 94 | 90 | 88 | 88 |
| | Xmin | 82 | 70 | 68 | 68 | 62 | 65 | 67 | 67 |
| Standing position 1 min | | 105.5±5.9 | 95.5±6.2 | 88.7±4.9 | 88.7±4.9 | 109.7±8.8 | 103.9±6 | 92.8±7 | 92.8±7 |
| | Xmax | 111 | 108 | 98 | 98 | 119 | 115 | 100 | 100 |
| | Xmin | 95 | 86 | 82 | 82 | 90 | 94 | 78 | 78 |
| 5 min | | 100.6±6.8 | 91.3±6 | 85.8±4.6 | 85.8±4.6 | 102.9±9.8 | 98.8±6 | 88.3±6.1 | 88.3±6.1 |
| | Xmax | 108 | 102 | 94 | 94 | 115 | 108 | 96 | 96 |
| | Xmin | 90 | 80 | 80 | 80 | 88 | 88 | 76 | 76 |
| 10 min | | 98.5±7.3 | 89.9±6.1 | 84.3±4.5 | 84.3±4.5 | 101.3±10.1 | 97.2±5.2 | 86.7±6.8 | 86.7±6.8 |
| | Xmax | 106 | 102 | 90 | 90 | 110 | 106 | 94 | 94 |
| | Xmin | 88 | 80 | 80 | 80 | 85 | 88 | 72 | 72 |
| Lying position 2 1 min | | 92.1±7 | 84.3±3.4 | 79.6±4.6 | 79.6±4.6 | 94.2±7.6 | 92.1±6.3 | 82.9±7.2 | 82.9±7.2 |
| | Xmax | 100 | 96 | 88 | 88 | 100 | 98 | 90 | 90 |
| | Xmin | 82 | 74 | 72 | 72 | 75 | 78 | 68 | 68 |
| 5 min | | 87.8±8 | 4.3±6.2 | 77±5.1 | 77±5.1 | 89.5±7.8 | 86.5±7.2 | 79.9±6.4 | 79.9±6.4 |
| | Xmax | 98 | 88 | 86 | 86 | 96 | 95 | 86 | 86 |
| | Xmin | 72 | 70 | 70 | 70 | 70 | 70 | 65 | 65 |
| 10 min | | 86.8±7.7 | 78.5±5.3 | 76±4 | 76±4 | 88±7.5 | 84.8±6.2 | 78.8±6.1 | 78.8±6.1 |
| | Xmax | 96 | 84 | 82 | 82 | 96 | 90 | 86 | 86 |
| | Xmin | 72 | 69 | 70 | 70 | 70 | 70 | 65 | 65 |

**Table 3. Mean values, standard deviations, the largest and smallest data values of systolic blood pressure during the orthostatic test in two (SOR and HOR) study groups before and after kinesitherapy, distributed classification by Thulesius**

| Parameter | Groups | SOR At beginning | $10^{th}$ Day | $1^{st}$ Month | $3^{rd}$ Month | HOR At beginning | $10^{th}$ Day | $1^{st}$ Month | $3^{rd}$ Month |
|---|---|---|---|---|---|---|---|---|---|
| Lying position 1 1 min | | 141.6±8.6 | 135.8±5.8 | 131.2±5.9 | 131.2±5.9 | 147.1±9.9 | 144.6±10 | 138.7±7 | 138.7±7 |
| | Xmax | 155 | 145 | 140 | 140 | 160 | 155 | 145 | 145 |
| | Xmin | 130 | 130 | 120 | 120 | 130 | 125 | 130 | 130 |
| 5 min | | 139.1±7.4 | 132±5.6 | 128.7±3.6 | 128.7±3.6 | 143.6±9.5 | 140.5±10.2 | 136.9±7.1 | 136.9±7.1 |
| | Xmax | 150 | 140 | 135 | 135 | 155 | 150 | 145 | 145 |
| | Xmin | 125 | 125 | 120 | 120 | 128 | 120 | 125 | 125 |
| 10 min | | 140±7.2 | 132±5.6 | 128.7±5.5 | 128.7±5.5 | 144±9.8 | 140.5±10.2 | 136.3±6.9 | 136.3±6.9 |
| | Xmax | 150 | 140 | 135 | 135 | 155 | 150 | 145 | 145 |
| | Xmin | 125 | 125 | 125 | 125 | 128 | 120 | 125 | 125 |
| Standing position 1 min | | 131.2±10 | 126.6±7.8 | 125±7.2 | 125±7.2 | 165.1±9.4 | 144±15.6 | 134.9±12.4 | 134.9±12.4 |
| | Xmax | 145 | 135 | 135 | 135 | 175 | 165 | 155 | 155 |
| | Xmin | 110 | 115 | 115 | 115 | 150 | 115 | 120 | 120 |
| 5 min | | 135.4±10.7 | 128.7±6.6 | 125.8±4 | 125.8±4 | 158.7±8.3 | 143.1±12.1 | 136.8±9.1 | 136.8±9.1 |
| | Xmax | 155 | 140 | 130 | 130 | 170 | 155 | 150 | 150 |
| | Xmin | 120 | 120 | 120 | 120 | 145 | 120 | 125 | 125 |
| 10 min | | 136.2±10.4 | 128.7±6.6 | 125.8±4 | 125.8±4 | 158±9.4 | 143.1±12.1 | 135.6±10.9 | 135.6±10.9 |
| | Xmax | 155 | 140 | 130 | 130 | 170 | 155 | 150 | 150 |
| | Xmin | 120 | 120 | 120 | 120 | 140 | 120 | 120 | 120 |
| Lying position 2 1 min | | 137.5±6.7 | 125.4±5.6 | 125±4.6 | 125±4.6 | 151.9±8.8 | 137.8±11.5 | 131.2±8.3 | 131.2±8.3 |
| | Xmax | 145 | 140 | 135 | 135 | 165 | 150 | 145 | 145 |
| | Xmin | 125 | 120 | 120 | 120 | 135 | 120 | 120 | 120 |
| 5 min | | 140±6.2 | 125±4.6 | 124.1±3.5 | 124.1±3.5 | 147.8±8.3 | 136.5±10.1 | 130.9±8.6 | 130.9±8.6 |
| | Xmax | 150 | 135 | 130 | 130 | 155 | 150 | 145 | 145 |
| | Xmin | 130 | 120 | 120 | 120 | 130 | 120 | 120 | 120 |
| 10 min | | 139.1±6.5 | 125±4.6 | 124.1±3.5 | 124.1±3.5 | 147.1±8.3 | 135.9±10.6 | 130.3±9.2 | 130.3±9.2 |
| | Xmax | 150 | 135 | 130 | 130 | 155 | 150 | 145 | 145 |
| | Xmin | 130 | 120 | 120 | 120 | 130 | 120 | 120 | 120 |

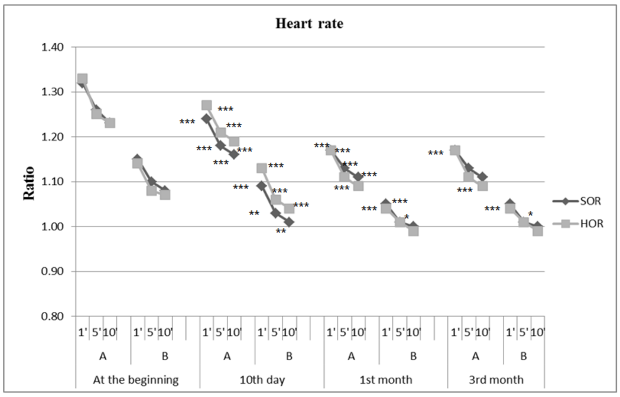

*Figure 1: Changes in average heart rate during the active orthostatic test given as the ratio of the obtained results and baseline values; A = active state; B = lying position. * P < 0.01, ** P <0.005, *** P < 0.0001 = significant difference compared with baseline values*

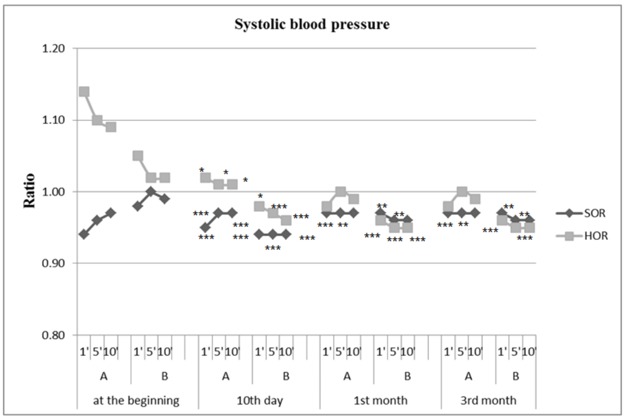

*Figure 2: Changes in mean SBP during active orthostatic test given as the ratio of the obtained results and baseline values; A = active state; B = lying positio. * P <0.01, ** P <0.005, *** P <0.0001 = significant difference compared with baseline values*
